# RNAi Screening Uncovers a Synthetic Sick Interaction between CtIP and the BARD1 Tumor Suppressor

**DOI:** 10.3390/cells11040643

**Published:** 2022-02-12

**Authors:** Hella A. Bolck, Sara Przetocka, Roger Meier, Christine von Aesch, Christina Zurfluh, Kay Hänggi, Vincent Spegg, Matthias Altmeyer, Michael Stebler, Simon F. Nørrelykke, Peter Horvath, Alessandro A. Sartori, Antonio Porro

**Affiliations:** 1 Institute of Molecular Cancer Research, University of Zurich, 8057 Zurich, Switzerland; hella.bolck@usz.ch (H.A.B.); sprzetocka@salk.edu (S.P.); vonaesch@imcr.uzh.ch (C.v.A.); zurfluh@imcr.uzh.ch (C.Z.); kay.hanggi@moffitt.org (K.H.); 2 Institute of Pathology and Molecular Pathology, University Hospital Zurich, 8091 Zurich, Switzerland; 3 Molecular and Cell Biology Laboratory, The Salk Institute for Biological Studies, La Jolla, CA 92037, USA; 4 Scientific Center for Optical and Electron Microscopy (ScopeM), ETH Zurich, 8093 Zurich, Switzerland; roger.meier@scopem.ethz.ch (R.M.); michael.stebler@scopem.ethz.ch (M.S.); simon.noerrelykke@scopem.ethz.ch (S.F.N.); 5 Department of Immunology, H. Lee Moffitt Cancer Center and Research Institute, Tampa, FL 33612, USA; 6 Department of Molecular Mechanisms of Disease, University of Zurich, 8057 Zurich, Switzerland; vincent.spegg@dmmd.uzh.ch (V.S.); matthias.altmeyer@uzh.ch (M.A.); 7 Synthetic and System Biology Unit, Biological Research Center (BRC), 6726 Szeged, Hungary; horvath.peter@brc.hu; 8 Institute for Molecular Medicine Finland, University of Helsinki, 00014 Helsinki, Finland

**Keywords:** CtIP, BARD1, BRCA1, synthetic lethality, replication stress, DNA damage

## Abstract

Human CtIP is best known for its role in DNA end resection to initiate DNA double-strand break repair by homologous recombination. Recently, CtIP has also been shown to protect reversed replication forks from nucleolytic degradation upon DNA replication stress. However, still little is known about the DNA damage response (DDR) networks that preserve genome integrity and sustain cell survival in the context of CtIP insufficiency. Here, to reveal such potential buffering relationships, we screened a DDR siRNA library in CtIP-deficient cells to identify candidate genes that induce synthetic sickness/lethality (SSL). Our analyses unveil a negative genetic interaction between CtIP and BARD1, the heterodimeric binding partner of BRCA1. We found that simultaneous disruption of CtIP and BARD1 triggers enhanced apoptosis due to persistent replication stress-induced DNA lesions giving rise to chromosomal abnormalities. Moreover, we observed that the genetic interaction between CtIP and BARD1 occurs independently of the BRCA1-BARD1 complex formation and might be, therefore, therapeutical relevant for the treatment of BRCA-defective tumors.

## 1. Introduction

Faithful transmission of genetic information to daughter cells is a central process for the maintenance of genome stability and the suppression of cancer and relies on accurate and complete DNA replication during S-phase. A variety of DNA lesions and structural impediments resulting from both exogenous and endogenous sources can obstruct replication fork progression leading to replication stress, a potent driving force of genomic instability and tumorigenesis [1]. Because of the serious implications of replication stress, genome duplication requires the precise coordination of DNA replication and repair processes [2]. Intriguingly, proteins commonly involved in homology-directed repair (HDR) of DNA double-strand breaks (DSBs) also protect stalled replication forks [3]. For instance, there is ample evidence that loss of BRCA1 and BRCA2 results in nucleolytic degradation of nascent DNA at stalled forks, ultimately giving rise to chromosomal instability [4].

Human CtIP was first recognized for its essential role in promoting DNA end resection, thereby committing DSBs to HDR [5]. More recent findings described additional functions of CtIP in response to replication stress, including efficient replication fork restart, suppression of new origin firing and promoting common fragile site stability [6,7]. Consistently, isolation of proteins on nascent DNA (iPOND) analysis suggested that CtIP associates with unperturbed replication forks [8]. Finally, we have recently proposed that CtIP limits excessive fork degradation in a BRCA1-independent manner [9]. This was rather unexpected as a direct interaction between the tandem BRCT repeats of BRCA1 and phosphorylated CtIP was reported to promote efficient DSB resection and subsequent HDR [10,11,12,13]. However, several studies have challenged this notion suggesting that complex formation with BRCA1 is largely dispensable for CtIP-mediated resection and ensuing HDR [14,15,16].

To gain insights into the molecular network collaborating with CtIP in genome stability maintenance, we screened for factors exhibiting an aggravating, synthetic sick or lethal (SSL) genetic interaction with CtIP. SSL interactions occur if two otherwise viable single gene disruptions lead to cell death or severely impaired cell growth when combined [17]. In this study, we conducted an image-based high-content RNA interference (RNAi) screen to identify genes required to sustain proliferation of SV40-immortalized MRC5 cells conditionally depleted of CtIP. Besides other DNA damage response (DDR) proteins, our analysis pipeline revealed a significant SSL interaction between CtIP and BARD1, the heterodimeric and obligate binding partner of BRCA1. Dissecting the underlying mechanism, we observed that CtIP and BARD1 are involved in separate aspects of preventing and addressing DNA lesions arising from endogenous replication stress. We demonstrate that combined disruption of CtIP and BARD1 results in a synergistic increase in DNA damage signalling and apoptosis, most likely caused by persistent fork stalling at difficult-to-replicate regions. Moreover, we propose that elevated levels of replication stress-induced DNA lesions combined with the inherent HDR deficiency of cells co-depleted of CtIP and BARD1 accelerates the formation of gross chromosomal aberrations. Our study thus uncovers a previously unanticipated buffering relationship between CtIP and BARD1 to ensure faithful DNA replication and maintain genome stability and corroborate the significance of the genetic interaction between CtIP and the BRCA1-BARD1 complex for tumor suppression.

## 2. Materials and Methods

### 2.1. Cell Culture

U2OS and U2OS Flp-In T-REx, HEK293T, and HEK293 cells were grown in DMEM supplemented with 10% fetal calf serum (FCS; GIBCO/Thermo Fisher Scientific, Waltham, MA, USA), 100 U/mL penicillin, and 100 mg/mL streptomycin (Sigma-Aldrich, St. Louis, MO, USA). MRC5^shCtIP^ and MRC5^shLacZ^ cells [18] were grown in DMEM supplemented with 10% Tet-System Approved FCS (GIBCO/Thermo Fisher Scientific), 100 U/mL penicillin, 100 µg/mL streptomycin, 5 µg/mL blasticidin (InvivoGen/LabForce, Muttenz, Switzerland), and 250 µg/mL Zeocin™ (InvivoGen/LabForce). To induce shRNA expression, cells were treated with 1 µg/mL doxycycline (Dox; Clontech/Takara, Kusatsu, Japan) as indicated. The Flp-In™ T-REx™ system (Invitrogen/Life Technologies, Carlsbad, CA, USA) was used to generate U2OS cell lines inducibly expressing siRNA-resistant GFP-CtIP and GFP-BARD1 [19]. In brief, single clones resistant to 250 µg/mL hygromycin B (InvivoGen/LabForce) and 12.5 µg/mL blasticidin were selected and screened for inducible GFP-BARD1 expression by immunofluorescence microscopy and immunoblotting.

U2OS Flp-In T-REx cell lines inducibly expressing siRNA-resistant RFP-FLAG-BARD-wt and the L44R mutant variant were a kind gift from Jo Morris (University of Birmingham, U.K.) [20]. To induce expression of BARD1, cells were treated with 1 µg/mL Dox as indicated. Hydroxyurea (HU) and camptothecin (CPT) were obtained from Sigma-Aldrich. dNTP analogs CldU and IdU (Sigma-Aldrich) were used as indicated.

### 2.2. RNAi Image-Based Screening and Data Analysis

MRC5^shCtIP^ cells were treated with 1 µg/mL Dox or left untreated (control) for 48 h prior to reverse transfection with a custom Silencer^®^ Select siRNA library (Ambion, Austin, TX, USA) targeting 207 human genes (Table S4). The library is comprised of three independent siRNA oligonucleotides per target arrayed alongside controls in 384-well plates (Greiner bio-one, Tokyo, Japan). All liquid handling was completed using an EL406 microplate washer and dispenser (BioTek, Winooski, VT, USA). RNAi screens were performed in three biological replicates, each consisting of two technical replicates. In brief, 0.8 pmol siRNA dissolved in 5 µL nuclease-free water were pre-spotted in each well of an assay plate. To this 25 µL DMEM (without supplements) containing 0.05 µL Lipofectamine RNAiMax (Invitrogen, Waltham, MA, USA) was added. The mix was incubated for 1 h at RT before dispensing 180 cells resuspended in 50 µL DMEM supplemented with 10% FCS, 100 U/mL penicillin, and 100 µg/mL streptomycin leading to a final concentration of 10 nM siRNA per well. For MRC5^shCtIP^ cells that had been exposed to Dox before, the cell culture media remained supplemented with 0.1 µg/mL Dox throughout the screen. Upon reverse transfection, cells were incubated for 6 days, followed by formaldehyde fixation and Hoechst staining (bisBenzimide H 33342; Sigma-Aldrich). The cells were imaged on two coupled ImageXpress micro (IXM) HCS microscopes (Molecular Devices) acquiring 9 images per well with a Nikon CFI S Fluor 10× objective. Finally, quantitative image analysis was carried out using CellProfiler (Broad Institute [21], Cambridge, MA, USA) and Matlab (The MathWorks, Natick, MA, USA) to assess the number of nuclei per well.

The numbers of nuclei per well were square root transformed and within-plate normalization was performed to median values of negative control conditions (percentage of control), i.e., mock-transfected cells or cells treated with non-targeting siRNAs. The strictly standardized mean difference (SSMD) was used to measure the magnitude of difference in cell viability between the cytotoxic positive control (siRNA against kinesin family member 11) and the negative controls as a means for quality control [22]. Target genes whose depletion induced cytotoxicity reducing cell numbers to more than 20% of the overall median cell number were excluded from further analysis. To identify negative synthetic genetic interactions with CtIP, a multiplicative model was applied and an interaction score, termed the (ε)-score, was computed. Based on the assumption that the combined effect of two individual perturbations (V_ab_) will be the product of their individual effects (V_a_ and V_b_), the ε score determined the difference between the observed phenotype and the effect that would be expected if the two genes do not interact [23]. These ε scores were used as a basis of hit ranking applying the redundant siRNA activity (RSA) algorithm. First, siRNAs were ranked according to their ε score after which the RSA method was used to assign cumulative hypergeometric probability (*p*) values to each target gene (Table S4) [24]. A *p*-value cut-off of *p* < 0.05 was chosen to determine a set of candidate hits.

### 2.3. Antibodies

A complete list of all primary antibodies used in this study can be found in Table S1. Secondary HRP-conjugated anti-mouse and anti-rabbit antibodies for immunoblotting were from GE-Healthcare (Chicago, IL, USA). Secondary AlexaFluor-488, -594 and -647-conjugated anti-mouse and anti-rabbit antibodies and Cy3-conjugated anti-rat antibody for immunofluorescence microscopy, flow cytometry and DNA fiber analysis were from Invitrogen and Jackson ImmunoResearch (West Grove, PA, USA), respectively.

### 2.4. siRNA Transfections and Sequences

All siRNA duplexes used in this study were purchased from Ambion and are listed in Table S2. siRNA oligos were transfected at a final concentration of 10 nM using Lipofectamine RNAiMax (Invitrogen) as indicated. For co-depletion experiments, the respective siRNAs were transfected at a final concentration of 10 nM + 10 nM of each oligonucleotide and the total amount of oligonucleotides was kept equal by transfecting a non-targeting siRNA (CTNL) in the single depletion samples.

### 2.5. Plasmids and Cloning

GFP-tagged human BARD1 expression constructs were kindly provided by Xiaochun Yu (University of Michigan Medical School) [25] and Richard Baer (Columbia University) [26]. Site-directed mutagenesis was used to introduce the non-coding mutations for siBARD1 resistance and the BARD1 single amino acid substitution mutations and was performed using the Expand Long Template PCR System (Roche, Basel, Switzerland). BARD1 sequences were subcloned using PCR into pcDNA5/FRT/TO GFP expression vector. DNA primers used for cloning and sequencing were obtained from Microsynth AG (Balgach, Switzerland) and are listed in Table S3. pEGFP-C1 plasmids containing CtIP wild-type were described previously [18]. Plasmids were transfected either by using the standard calcium phosphate method or by FuGENE 6 (Roche) according to the manufacturer’s instructions. 

### 2.6. Immunoblotting and Immunoprecipitation Assays

If not specified otherwise, cell extracts were prepared in Laemmli buffer (4% SDS, 20% glycerol, 120 mM Tris-HCl pH 6.8). Proteins were resolved by SDS–PAGE and transferred to the nitrocellulose membrane. Immunoblots were performed by using the appropriate antibodies and proteins were visualized using the ECL detection system (Western Bright^TM^, Advansta, San Jose, CA, USA) imaging on a FusionSolo (Witec AG, Heitersheim, Germany). U2OS Flp-In T-REx cell lines inducibly expressing siRNA resistant RFP-FLAG-BARD1-wt and the L44R mutant variant were lysed in RIPA buffer (50 mM Tris-HCl (pH 7.5), 1% NP-40, 0.25% sodium-deoxycholate, 150 mM NaCl, 1 mM EDTA, 0.1% SDS, protease inhibitors (1 mM benzamidine and 0.1 mM PMSF)), subjected to benzonase treatment (10 U Benzonase^®^ (Roche)) for 30 min at 4 °C, cleared by centrifugation (14,000 rpm) and immunoprecipitated using the M2-agarose anti-FLAG resin (Sigma-Aldrich) overnight at 4 °C. Immunocomplexes were stringently washed four times with RIPA buffer followed by one wash with TEN100 buffer (0.1 mM EDTA, 20 mM Tris-HCl, pH 7.4, 100 mM NaCl), boiled in SDS-sample buffer and analyzed by SDS-PAGE followed by immunoblotting.

### 2.7. Flow Cytometry Analysis

Where indicated, cells were transfected with siRNA as described above, and the knockdown was allowed to persist for 48 h. Cell cycle in combination with γH2AX analysis was carried out as previously described [27]. Shortly, cells were treated as described in the figure legends, harvested by trypsinization, and fixed using 4% formaldehyde in PBS (*w*/*v*). For γH2AX analyses, cells were incubated in 0.5% saponin/1% BSA/PBS containing the primary antibodies for 2 h at RT, followed by incubation with secondary antibodies and staining of the DNA with DAPI (DNA content). Fluorescence was measured on LSR II Fortessa (BD Bioscience, Franklin Lakes, NJ, USA) and analyzed with FlowJo X (Tree Star, Ashland, OR, USA).

To assess apoptosis, U2OS were transfected with siRNA as described above and re-plated into 6-well tissue culture plates. On the indicated days, cells were harvested by trypsinization. Cells were collected in polysterene FACS tubes and washed with annexin binding buffer (10 mM HEPES/NaOH, 140 mM NaCl, 2.5 mM CaCl_2_ (pH 7.4)). After washing, cells were stained in 50 µL annexin binding buffer, including 5 µL annexin V-FITC (eBioscience, San Diego, CA, USA) and propidium iodide (1 μg/mL; Thermo Fisher, Waltham, MA, USA) for 20 min at RT. Cells were then washed with annexin binding buffer before analysis by flow cytometry with Attune (Thermo Fisher) and analyzed with FlowJo X (Tree Star).

For detection of apoptotic cells by cleaved caspase-3 immunofluorescence FACS, U2OS cells were transfected with indicated siRNAs. The next day, cells were plated on 6-well plate and processed as described previously [28] 48 or 96 h post-transfection. Briefly, medium was collected in 15 mL falcon tubes, cells were harvested by trypsinization and centrifuged at 1000 rpm for 5 min at 4 °C. Pellets were washed once with cold PBS and fixed in 4% formaldehyde for 10 min at RT. Next, pelleted cells were resuspended in 100 µL PBS and 900 µL of −20 °C methanol was added drop-wise while vortexing. Fixed cell were stored at −4 °C until ready for immunostaining. Cells were then washed with PBS and blocked for 30 min at RT in blocking buffer (10% FCS, 0.5% NP-40, 0.5% saponin in PBS). After blocking pellets were resuspended in 200 µL of primary antibody dilution in blocking buffer. After 2 h incubation at RT, cells were washed with PBS and incubated for 1 h in 200 µL of secondary antibody dilution. Finally, cells were washed with PBS, resuspended in 500 µL PBS and cleaved caspase-3 positive cells were analyzed on Attune NxT Flow Cytometer (Thermo Fisher).

### 2.8. Immunofluorescence Microscopy

U2OS cells grown on coverslips were either fixed directly in 4% formaldehyde in PBS (*w*/*v*) and permeabilized in 0.3% Triton X-100/PBS or pre-extracted for 5–10 min on ice before fixation in formaldehyde for 15 min as described previously [5]. After incubation with the indicated primary and appropriate secondary antibodies, coverslips were mounted with Vectashield^®^ (Vector Laboratories, Burlingame, CA, USA) containing DAPI and sealed. Images were acquired on a Leica DMI 6000 fluorescence microscope at 63× magnification. Images were analyzed using customized CellProfiler pipelines [21]. 53BP1 foci were identified and related to obtain a per-nucleus aggregate measurement (number of 53BP1 foci per nucleus). For complementation experiments, nuclei were identified and classified according to the per-nucleus staining intensities measured in the green channel (expression of GFP-tagged fusion proteins). The number of 53BP1 foci in GFP-positive nuclei was determined. 

### 2.9. Quantitative Image-Based Cytometry (QIBC)

QIBC was performed as previously described [9]. In brief, U2OS and U2OS Flp-In T-REx cells transfected with indicated siRNAs were grown on glass coverslips. At 48 h post-transfection cells were cultivated with EdU (10 µM) for 20 min and fixed with 4% formaldehyde in PBS for 15 min at RT. The samples were washed twice with 3% BSA in PBS, permeabilized in 0.5% Triton X-100 in PBS for 20 min, and washed again with 3% BSA/PBS. The Click-it EdU reaction for cell cycle analysis was performed according to the manufacturer’s instructions prior to incubation with detergent solution (0.1% Triton X-100, 0.02% SDS in PBS) for 5 min at RT. Coverslips were then blocked for 30 min in blocking solution (3% FCS in 3% BSA/PBS). The samples were subsequently incubated in blocking solution containing primary antibodies for 2 h at RT, washed with PBS, and incubated with secondary antibodies for 1 h at RT. Finally, the coverslips were stained with DAPI (1 µg/mL) for 15 min at RT and mounted on glass slides with Mowiol-based mounting (Mowiol 4.88/Glycerol/Tris). Images were obtained using automated multichannel wide-field microscopy as described previously [29] on an Olympus ScanR Screening System with UPLSAPO 20× objective (NA 0.75). Image analysis was carried out using the built-in Olympus ScanR Image Analysis Software Version 2.5.1. Dynamic background correction was applied to all images, the DAPI signal was used to identify individual nuclei by an intensity-based object detection module, and foci segmentation was performed using a spot-detection module. Obtained object and associated sub-object and fluorescent intensities were exported into TIBCO Spotfire software (TIBCO, Somerville, MA, USA), which was used to generate color-coded scatterplots. For visualizing discrete data in scatterplots (e.g., foci numbers), mild jittering (random displacement of data points along the discrete data axes) was applied in order to demerge overlapping data points. 

### 2.10. Cell Proliferation and Viability Assays

For drug hypersensitivity assays, cells transfected with siRNA were seeded in triplicates at a density of 500 or 1000 cells/well in 96-well plates 24 h post-transfection or after administration of 1 µg/mL Dox (MRC5^shRNA^). Cells were exposed to the indicated doses of CPT at 24 h after plating and grown for 4 days at 37 °C. To measure cell viability, CellTiter-Blue^®^ reagent (Promega, Madison, WI, USA) was added on the last day, cells were incubated at 37 °C for 4 h, and then fluorescence was measured at 560/590 nm using a SpectraMax^®^ i3 Microplate Reader (Molecular Devices). For screen validation, 500 or 1000 U2OS cells were reverse transfected with 10 nM + 10 nM of the indicated siRNAs in a 96-well plate in triplicates. Cell proliferation was allowed for 6 days before addition of CellTiter-Blue^®^ reagent (Promega).

### 2.11. Colony Formation Assay

MRC5^shCtIP^ cells were transfected with the indicated siRNA using Lipofectamine RNAiMax (Invitrogen). On the next day, cells were re-plated in triplicates into two sets of 6-well plates at low cell dilutions. At 48 h post transfection, one set of 6-well plates was exposed to 1 µg/mL Dox to induce shCtIP expression while the other one was kept untreated as a control. Cells were cultured for 10 days to allow the formation of colonies. Subsequently, colonies were fixed and stained in crystal violet/ethanol (0.5%/20%) solution. Colonies reaching a minimum of 50 cells were counted and the number of colonies without Dox was set to be 100% for each siRNA transfection in order to determine the effect of CtIP co-depletion.

To assess clonogenic survival, U2OS Flp-In T-REx cell lines were seeded into 6 cm dishes and the day after transfected with indicated siRNA using Lipofectamine RNAiMax (Invitrogen). A total of 6 h later the expression of GFP-CtIP-wt, RFP-Flag-BARD1-wt or RFP-Flag-BARD1-L44R was induced with the addition of doxycycline (1 µg/mL). A total of 24 h post-transfections cells were re-plated in triplicates into 6-well plates at low cell dilutions (see figure legends for details). After 14 days of growth, cells were fixed with crystal violet solution (0.5% crystal violet, 20% ethanol (*w*/*v*)). Plates were scanned and survival was analyzed with the ImageJ Plugin Colony Area using the parameter colony intensity as read-out [30].

### 2.12. HDR Reporter Assay

U2OS-TLR cells [31] were transfected with siRNA and after 6 h transfected with the *I-SceI*-expression plasmid and the exogenous donor template containing the missing part of eGFP. A total of 72 h after siRNA transfection, cells were collected by trypsinization, and analyzed by flow cytometry using an LSRII Fortessa (BD Bioscience) as described previously [31,32]. Data analysis was performed using FlowJo X (Tree Star) to assess the percentages of eGFP-positive cells depicting the efficiency of homologous recombination.

### 2.13. DNA Fiber Spreading

DNA fiber analyses were carried out as described previously [33]. For measuring sister fork asymmetry, cells were labeled with CldU (33 μM) for 30 min followed by exposure to 2 mM HU for 2 h and chased with IdU (340 μM) for 40 min before harvesting in PBS. Cells were lysed (lysis buffer: 200 mM Tris-HCl (pH 7.4), 50 mM EDTA, 0.5% SDS) and DNA fibers stretched onto glass slides and fixed in methanol:acetic acid (3:1, Merck). After rehydration in PBS, these were denatured with 2.5 M HCl for 1 h, washed with PBS and blocked with 2% BSA in PBS containing 0.1% Tween 20 for 30 min. The newly replicated CldU and IdU tracks were immunostained using anti-BrdU primary and appropriate secondary antibodies. Coverslips were mounted using Antifade Gold (Invitrogen). Images were acquired on a Leica DMI 6000 fluorescence microscope and analyzed using Fiji software [34]. Statistics were calculated in Graph Pad Prism (GraphPad Software Inc., San Diego, CA, USA).

### 2.14. Pulsed Field Gel Electrophoresis (PFGE)

PFGE was essentially carried out as described previously [35]. In brief, cells were collected by trypsinization, and agarose plugs containing 600,000 cells were prepared. The plugs were then incubated in lysis buffer (100 mM EDTA, 1% (*w*/*v*) sodium lauryl sarcosyne, 0.2% (*w*/*v*) sodium deoxycholate, 1 mg/mL proteinase K) at 37 °C for 72 h and then washed four times in washing buffer (20 mM Tris-HCl (pH 8.0), 50 mM EDTA) for 30 min before loading them onto an agarose gel. Electrophoresis was performed for 21 h at 14 °C in 0.9% (*w*/*v*) pulsed field-certified agarose (Biorad, Hercules, CA, USA) containing TBE-Buffer using a CHEF-DR III PFGE system (Biorad) and the running protocol as described in Zellweger et al. [36].

### 2.15. Metaphase Spreads

Metaphase spreads were prepared as described previously [18]. In brief, HEK293 cells were incubated with 0.1 µg/mL colcemid for 3 h and harvested by trypsinization. Cell pellets were resuspended in 5 mL of hypotonic potassium chloride (75 mM) solution and incubated for 10 min at 37 °C for swelling. Cells were fixed once with 5% acetic acid for 3 min and then twice with ethanol-acetic acid (3:1) for 10 min. Fixed cells were gently resuspended in fixative solution to yield an optimal cell density and dropped onto glass slides before staining with Giemsa or DAPI. Phase contrast or fluorescent images were acquired using an Olympus IX83 inverted or a Leica DMI 6000 fluorescence microscope, respectively, at 63× magnification.

### 2.16. Statistical Analysis

Statistical analyses were performed using GraphPad Prism, and statistical tests are reported in the figure legends. If not indicated otherwise, each experiment was repeated at least three times. In all cases: ns—not significant (*p* > 0.05); * *p* ≤ 0.05; ** *p* ≤ 0.01; *** *p* ≤ 0.001; **** *p* ≤ 0.0001.

## 3. Results

### 3.1. RNAi Screening Unveils a Negative Genetic Interaction between CtIP and BARD1

**Figure 1:**
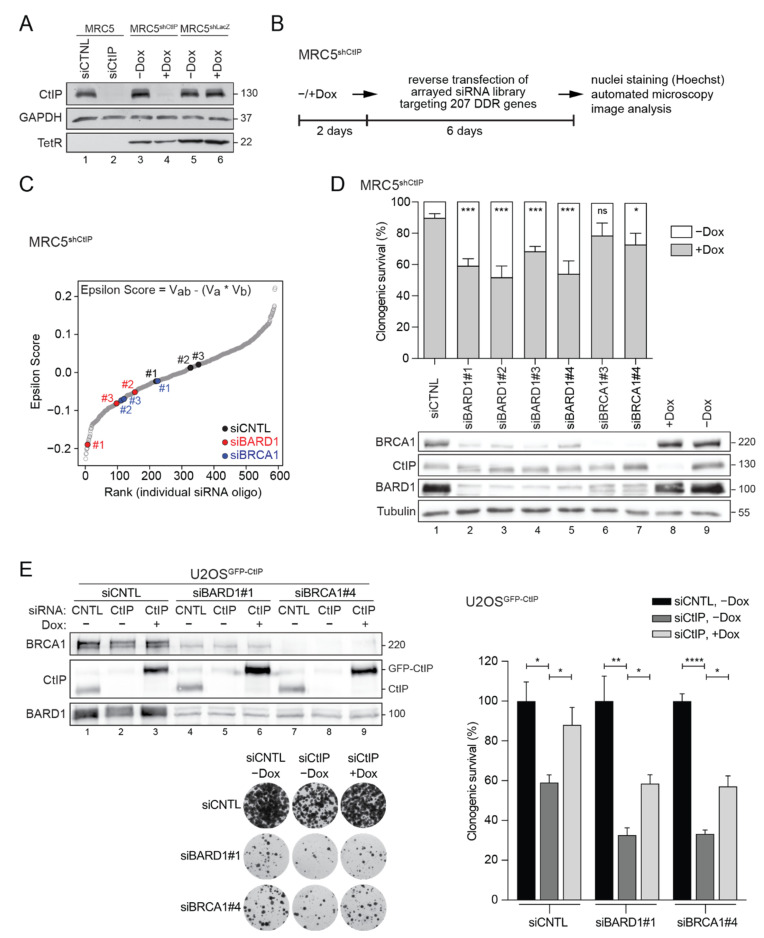
Identification of a synthetic sick genetic interaction between CtIP and BARD1. (**A**) SV40-transformed MRC5 cells were transfected with CtIP or control (CNTL) siRNA for 48 h. MRC5 cells stably expressing doxycycline (Dox)-inducible shRNA against CtIP (MRC5^shCtIP^) or LacZ (MRC5^shLacZ^) were cultivated in the absence or presence of Dox (1 µg/mL) for 48 h. Whole-cell lysates were prepared and subjected to immunoblotting using the indicated antibodies. (**B**) Schematic outline of the RNAi imaged-based screen to investigate synthetic genetic interactions with CtIP. (**C**) Genetic interactions were defined in terms of deviation (Epsilon score) from the expectation that the combined effect on cell viability (V) of two gene disruptions (a and b) will be the product of their individual effects (as determined by the formula). A ranking of Epsilon scores for all target siRNAs is displayed. Scores for three individual siRNAs against BARD1, BRCA1 and non-targeting siRNAs (CNTL) are highlighted. (**D**) MRC5^shCtIP^ cells were transfected with the indicated siRNAs and subjected to colony formation assay. Upper panel, Bars represent the reduction in cell viability upon CtIP co-depletion by Dox-inducible shRNA compared to siRNA-mediated knockdown of BARD1 or BRCA1 alone. Data represent the mean ± s.e.m. (*n* ≥ 3). Statistical significance was calculated with unpaired *t*-test. * *p* value ≤ 0.05, *** *p* value ≤ 0.001, ns *p* value > 0.05. Lower panel, Whole-cell lysates of the corresponding samples were subjected to immunoblotting using the indicated antibodies. (**E**) U2OS cells inducibly expressing GFP-CtIP were transfected with indicated siRNAs. At 6 h post transfection cells were treated with Dox (1 µg/mL). A total of 24 h later, cells were plated into 6-well plates at four densities (125, 250, 500 and 1000 cells per well), and grown for 14 days. Left panel, Knock-down efficiencies and GFP-CtIP induction were analyzed by immunoblot of the whole-cell lysates. Right panel, Colonogenic survival was determined by quantifying the colony intensity of siCtIP-transfected cells relative to siCTNL-transfected cells. Data, mean ± s.e.m. (*n* = 3). Statistical significance was calculated with unpaired *t*-test. * *p* value ≤ 0.05, ** *p* value ≤ 0.01, **** *p* value ≤ 0.0001. Representative images of clonogenic assays of the indicated U2OS^GFP-CtIP^ cells (1000 cells/well density) are shown.

Gene interaction networks can predict functional relationships between proteins and their underlying biological pathways [37,38,39]. Homologous recombination (HR) is an evolutionarily conserved process that plays a prime role in maintaining genome stability by repairing DSBs and preserving the integrity of stalled replication forks. HR genes are essential in mammals and their knockout often results in early embryonic lethality [40]. Partial loss-of-function of HR genes can result in genomic instability and the accumulation of mutations, ultimately driving tumorigenesis. We have recently found that CtIP and BRCA1, two HR factors, synergize to preserve genome stability upon replication stress [9]. To confirm this finding and eventually uncover novel synthetic genetic interactions between CtIP and other DDR proteins, we performed an image-based, combinatorial RNAi screen in MRC5^shCtIP^ cells stably expressing doxycycline (Dox)-inducible shRNA against CtIP [18]. CtIP knockdown efficiency in MRC5^shCtIP^ cells was comparable to that achieved by transfecting CtIP siRNA (Figure 1A and Figure S1A), both causing nearly identical hypersensitivity to camptothecin (CPT) (Figure S1A), a well-established phenotype of CtIP-deficient cells [5]. We screened a custom-made siRNA library targeting 207 genes annotated to key DDR-related pathways (Figure 1B and Table S4) to identify those that conferred a synthetic growth defect when combined with CtIP deficiency. To quantify non-epistatic genetic interactions, we applied a multiplicative model and determined an interaction score (ε) for every target siRNA (Figure 1C). We defined *bona-fide* genetic interactions in terms of deviation from the expectation that the combined effect following depletion of CtIP and each selected DDR factor on cell survival is the mere additive product of their individual effects. Pearson correlation coefficients revealed suitable data reproducibility between three biological replicates (Figure S1B). Image and data analysis indicated 18 candidate genes potentially exhibiting a negative genetic interaction with CtIP (RSA *p*-value cut-off of 0.05) (Figure S1C). BRCA1-associated RING domain protein 1 (BARD1) was among the strongest hits (RSA *p*-value 0.0175), with all three independent siRNAs used in the screen reproducibly reducing cell growth in a CtIP-deficient background, as denoted by negative epsilon scores (Figure 1C and Figure S1C). Notably, BRCA1 scored just slightly above the cut-off (RSA *p*-value 0.055) (Figure 1C and Figure S1C), suggesting that a functional BRCA1-BARD1 complex is important for cell viability in cells expressing critically low levels of CtIP. We validated the SSL phenotype between CtIP and BARD1 by colony formation assays in MRC5^shCtIP^ cells individually transfected with four different BARD1-targeting siRNAs, meanwhile knockdown of BRCA1 only led to a minor reduction in clonogenic survival (Figure 1D). Consistently, double knockdown of CtIP and BARD1 or BRCA1 in U2OS cells stably expressing doxycycline-inducible GFP-CtIP, significantly decreased cell viability that was partially rescued upon induction of CtIP expression (Figure 1E). However, given the established interdependence of BRCA1 and BARD1 protein stability [41], as observed in our western blots (Figure 1D,E), CtIP cannot fully compensate for the lack of the BRCA1-BARD1 complex. 

### 3.2. Prolonged Downregulation of CtIP and BARD1 Results in DNA Damage-Induced Apoptosis

**Figure 2:**
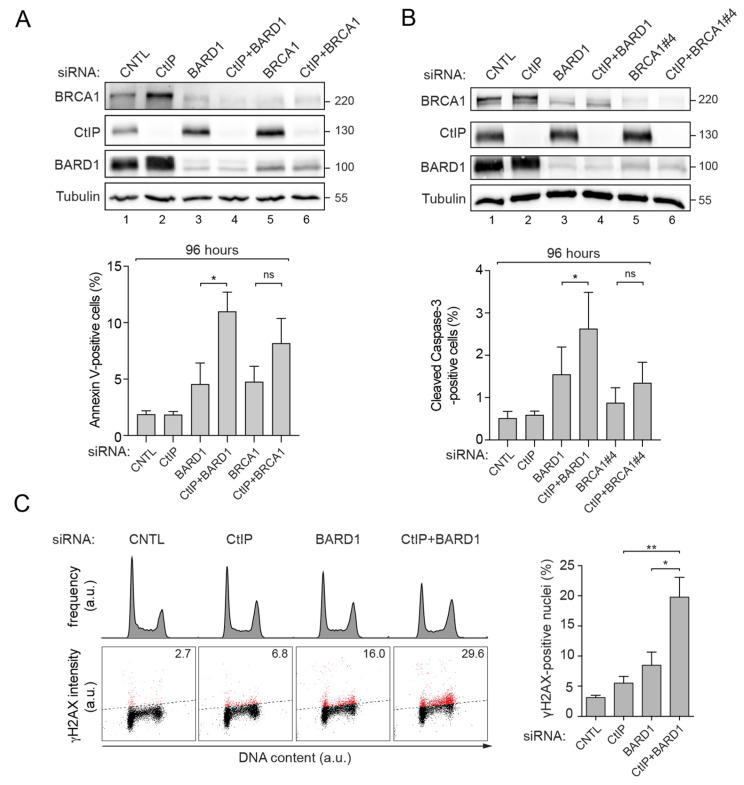
Concomitant loss of CtIP and BARD1 triggers apoptosis by DNA damage. (**A**,**B**) U2OS cells transfected with the indicated siRNA for 48 h were harvested and induction of apoptosis was determined by annexin V (**A**) or cleaved caspase-3 (**B**). Statistical significance was calculated with unpaired *t*-test. * *p* value ≤ 0.05, ns *p* value > 0.05. (**C**) Same cells as in (**A**) were fixed, permeabilized and immunostained with anti-γH2AX antibody along with DAPI before analysis by flow cytometry. Quantification gates were established in samples transfected with siCNTL. Percentages indicate mean ± s.e.m. of γH2AX-positive cells (*n* 3). Statistical significance was calculated with unpaired *t*-test. * *p* value ≤ 0.05, ** *p* value ≤ 0.01.

To delineate the underlying mechanism associated with reduced viability of cells deficient for both CtIP and BARD1, we measured apoptotic cell death by annexin V staining or caspase-3 cleavage at two- and four-days post-siRNA transfection. Co-depletion of CtIP and BARD1 provoked a significant, two-fold increase in the number of cells undergoing apoptosis after 96 h (Figure 2A,B), but not 48 h (Figure S2A,B). We speculated that due to the crucial roles of CtIP and BARD1 in HR, the accumulation of unrepaired DSBs over time might be primarily responsible for the observed cytotoxicity and apoptosis.

To test this hypothesis, we first monitored ATM autophosphorylation levels by Western blotting, considered as one of the earliest markers of DSB signalling [42]. ATM activation could already be observed in single-depleted cells and was further elevated in cells co-depleted of CtIP and BARD1 (Figure S2C). We next employed flow cytometry to measure phosphorylation of histone H2AX (γH2AX), a downstream target of ATM kinase and general DSB marker. Consistently, we observed that the percentage of γH2AX-positive cells synergistically increased upon simultaneous depletion of CtIP and BARD1 (Figure 2C). Moreover, cell cycle analyses showed that CtIP and BARD1 knockdown caused a gradual accumulation of cells in the G2/M phase (Figure 2C). 

The majority of spontaneous DSBs arise from single-strand breaks at stalled replication forks in response to dysregulated DNA replication [1]. Moreover, unresolved replication intermediates can be converted into DNA lesions when cells enter mitosis [43]. In human cells, 53BP1 was shown to recognize such sites of unrepaired DNA damage forming large nuclear bodies (NBs) upon subsequent entry into G1 to shield them against erosion [43]. Thus, to corroborate CtIP and BARD1 jointly suppressing replication-derived DSBs, we analyzed 53BP1 foci redistribution in U2OS cells subjected to CtIP and BARD1 siRNA transfection.

**Figure 3:**
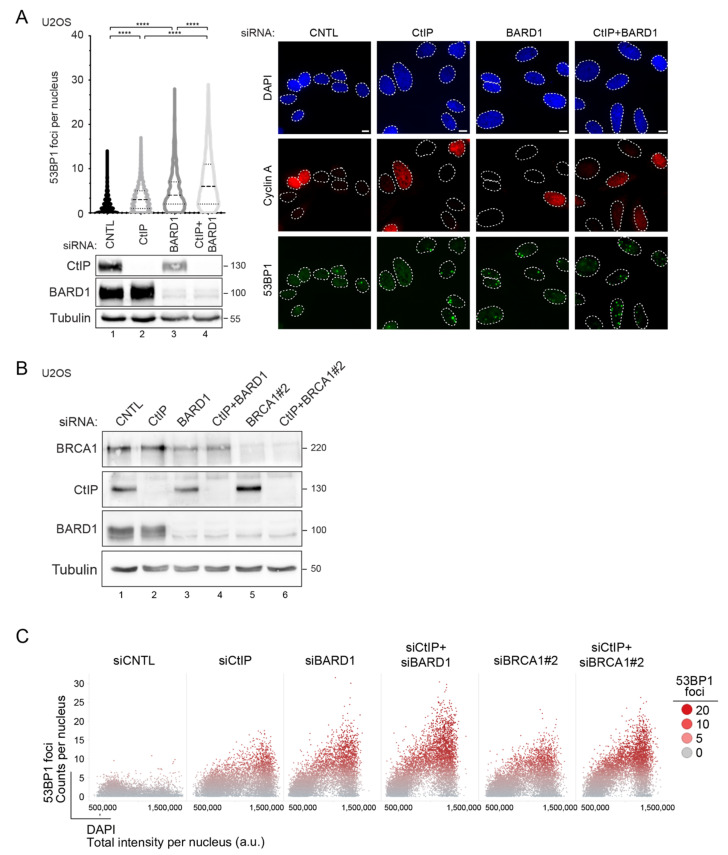
CtIP and BARD1 deficiency induces 53BP1 foci formation indicative of elevated replication stress. (**A**) U2OS cells transfected with the indicated siRNA for 48 h were fixed and immuno-fluorescence microscopy analysis was performed with anti-53BP1 antibodies (green) and anti-Cyclin A antibodies (red) along with DAPI staining. Left panel, Violin plots represent quantification of 53BP1 foci per nucleus. The dotted lines indicate the quartile of each distribution and the dashed line represents the median. At least 900 nuclei per knockdown condition (*n* ≥ 4) were assessed. Statistical significance was calculated with the Kolmogorov Smirnov test. **** *p* value ≤ 0.0001. Whole-cell lysates of the corresponding samples were subjected to immunoblotting using the indicated antibodies. Right panel, Representative microscopy images of U2OS cells. (**B**) U2OS cells transfected with the indicated siRNAs for 48 h were subjected to immunoblotting using the indicated antibodies. (**C**) Same cells as in (B) were fixed and stained for DNA content (DAPI) and 53BP1. The formation of 53BP1 foci was assessed in a cell-cycle-resolved manner by high-content quantitative image-based-cytometry (QIBC). Scatter plots depict the cell-cycle distribution of 53BP1 foci per nucleus. Each dot represents a single cell.

We observed that knockdown of CtIP, BARD1, or both factors together led to a progressive increase in the average number of 53BP1 NBs occurring in cyclin A-negative G1 cells (Figure 3A). 53BP1 foci accumulation in BARD1-depleted cells was rescued by stable expression of siRNA-resistant GFP-BARD1, ruling out siRNA-induced off-target effects (Figure S3A). We observed that the increase in the percentage of nuclei displaying supernumerary 53BP1 foci is not restricted to G1 cells but occurs throughout the cell cycle in both CtIP/BARD1 and CtIP/BRCA1 co-depleted cells (Figure 3B,C). Thus, we reasoned that combined disruption of CtIP and BARD1 leads to replication stress-induced DNA lesions that persist throughout the ensuing cell cycle due to impaired HR. Consistently, co-depletion of CtIP and BARD1 completely abolished homologous recombination repair efficiency as measured by GFP reporter assay [31] (Figure S3B).

Taken together, our data suggest that following combined depletion of CtIP and BARD1, cells experience elevated levels of unrepaired DSBs during DNA replication, triggering cell cycle arrest and cell death by apoptosis. 

### 3.3. BARD1-L44R Mutant Defective in BRCA1 Binding Recapitulates SSL Phenotype Observed between CtIP and BARD1

**Figure 4:**
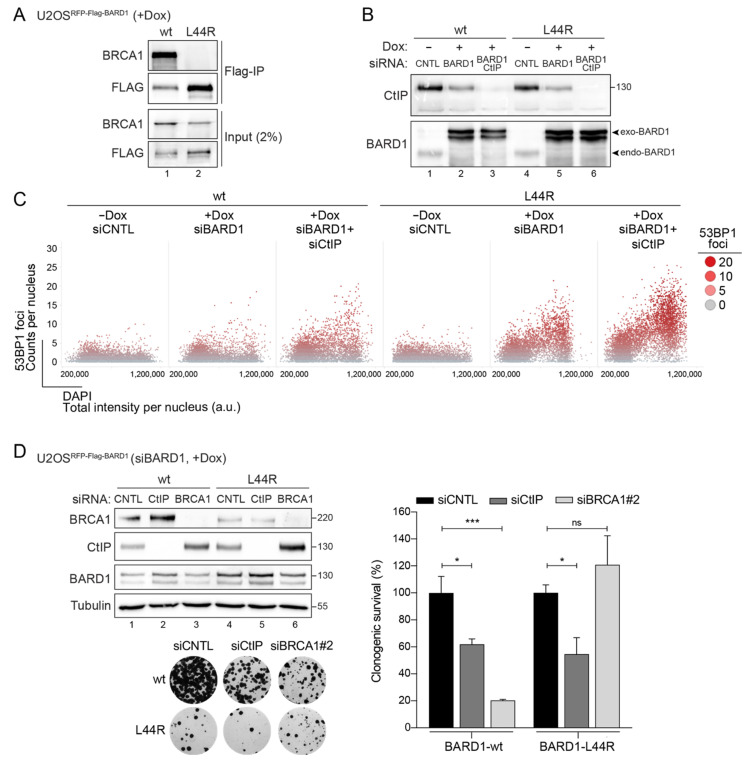
Synthetic lethality between CtIP and BARD1 occurs independently of the BARD1-BRCA1 interaction. (**A**) Whole-cell extracts from U2OS Flp-In T-REx cells stably expressing Dox-inducible RFP-FLAG BARD1 wt and L44R variant were subjected to FLAG-immunoprecipitation and subsequently to western-blot with the indicated antibodies. (**B**) Same cells as in (**A**) were transfected with siBARD1 in combination with either siCNTL or siCtIP for 48 h were subjected to immunoblotting using the indicated antibodies. (**C**) Same cells as in (**B**) were fixed and stained for DNA content (DAPI) and 53BP1. The formation of 53BP1 foci was assessed in a cell cycle-dependent manner by high-content quantitative image-based cytometry (QIBC). Scatter plots depict the cell cycle distribution of 53BP1 foci per nucleus. Each dot represents a single cell. (**D**) U2OS Flp-In T-REx cells stably expressing Dox-inducible RFP-FLAG-BARD1 wt and L44R variant were transfected with siBARD1 in combination with either siCNTL, siCtIP or siBRCA1. At 6 h post-transfection cells were treated with Dox (1 μg/mL). A total of 24 h later, cells were plated at low dilutions (500 and 1000 cells) into 6-well plates and grown for 14 days. Left panel, Protein levels were verified by immunoblotting of the whole-cell lysates. In addition, representative images of survival assay are depicted (1000 cells dilution). Right panel, Bar graph illustrates survival by calculating the colony intensity relative to siCNTL-transfected cells. Data, mean ± s.e.m. (*n* = 4). Statistical significance was calculated with unpaired *t*-test. * *p* value ≤ 0.05, *** *p* value ≤ 0.001, ns *p* value > 0.05.

The most widely accepted mechanistic explanation of negative synthetic genetic interactions comprises two genes functioning in parallel, mutually compensatory pathways, known as between-pathway SSL [17]. Physical interaction, on the other hand, is often interpreted to denote gene products functioning within the same pathway or protein complex [44,45]. However, recent findings revealed that multiple negative interactions occur between factors implicated in the same molecular pathway or even within one complex [46,47]. Intriguingly, DDR pathways represent a hotspot for within-pathway SSL interactions frequently connecting components of the same essential complex [46]. CtIP associates with BRCA1-BARD1 in a stable complex known as BRCA1-C [48,49]. To delineate whether the genetic interaction between CtIP and BARD1 relies on an intact BRCA1-BARD1 complex, we made use of the BARD1 L44R mutant that abrogates interaction with BRCA1 by introducing a large hydrophilic residue in the hydrophobic helix of BARD1 [50]. As shown previously, U2OS cells inducibly-expressing siRNA-resistant BARD1 L44R are defective in BRCA1-BARD1 heterodimer formation [51] (Figure 4A). When quantifying hundreds of individual cells by high content microscopy, we observed that, similar to BARD1 depletion, replacement of endogenous BARD1 with the L44R mutant led to an accumulation of replication-associated 53BP1 foci upon CtIP knockdown (Figure 4B,C). BARD1 L44R cells are clearly less viable than BARD1 wild-type cells, indicating that the integrity of the BRCA1-BARD1 complex is essential for cell viability. 

Nonetheless, we could consistently observe that survival of BARD1-L44R cells is significantly compromised by additional CtIP depletion, whereas BRCA1 co-depletion did not have any impact (Figure 4D). Mechanistically, one could envision a scenario where the BARD1-L44R is still recruited to damaged chromatin independently of BRCA1, possibly mediated by binding of its BRCT domain to poly-ADP-ribosylated (PAR) proteins, and, thus, to at least partially elicit HR repair and/or fork protection [25]. Consequently, decreased survival of BARD1-L44R cells following CtIP depletion could be due to either aggravated DSB repair defects or, as we postulate, excessive degradation of nascent DNA at stalled replication forks.

### 3.4. Combined Depletion of CtIP and BARD1 Fuels Replication Stress-Induced Genomic Instability

Besides their canonical function in DSB repair, it has been established that several HR proteins serve to protect stalled forks from nucleolytic processing and subsequent damage accumulation [3]. Interestingly, however, our recent study has implicated CtIP and BRCA1 to act in separate fork protection pathways [9]. 

**Figure 5:**
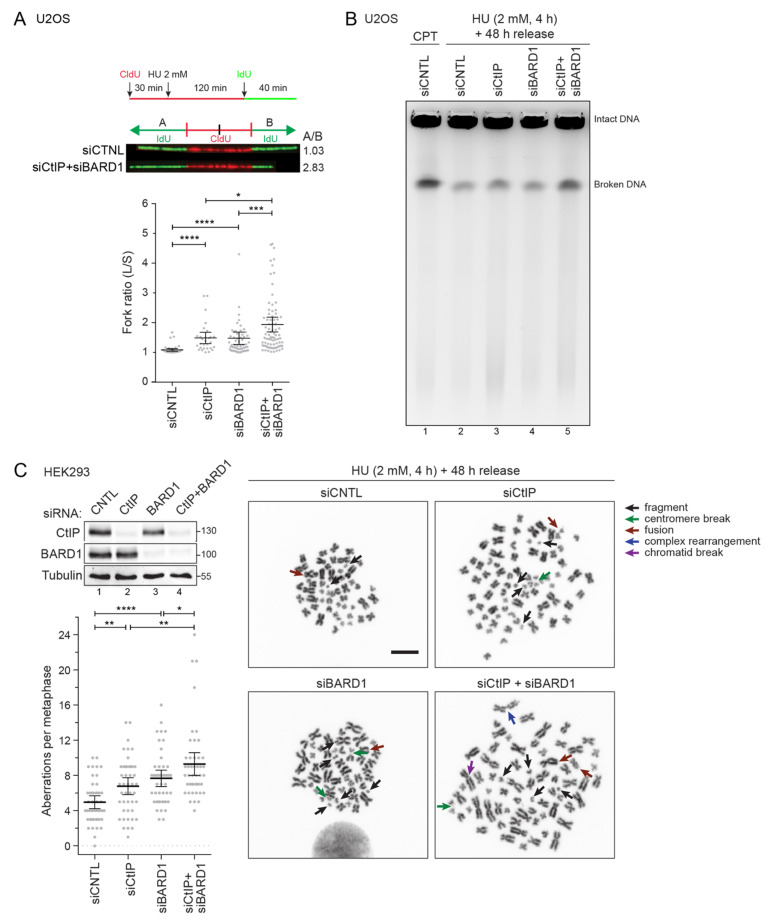
CtIP and BARD1 prevent accumulation of DSBs and chromosomal fragmentation in response to replication stress. (**A**) DNA fiber analysis of U2OS cells transfected with the indicated siRNAs for 48 h. Cells were pulsed with CldU, exposed to 2 mM HU for 2 h, and pulsed with IdU. Representative images are shown. Scatter plots indicate the ratios of left/right fork lengths of bidirectional replication forks traveling from a single origin. The lines denote mean ratios ± 95% confidence interval (*n* = 2). Statistical significance was calculated by Mann-Whitney test. * *p* value ≤ 0.05, *** *p* value ≤ 0.001, **** *p* value ≤ 0.0001. (**B**) U2OS cells were transfected with the indicated siRNA for 48 h and were treated with 2 mM HU for 4 h and subsequently released from HU for 48 h. Cells were harvested, and DNA fragments were separated by PFGE. In lane 1, siCNTL-transfected cells were treated with high-dose CPT (2 µM) for 4 h and immediately harvested for PFGE analysis. (**C**) HEK293 cells were transfected with the indicated siRNAs for 48 h, treated with 2 mM HU for 4 h, and released from HU for 48 h. Prior to harvest, cells were arrested in metaphase with 0.1 μg/mL colcemid for 3 h. Metaphase spreads were prepared according to standard protocols and stained with DAPI. Representative images are shown (right panel). Left panel, Aberrations from 30 metaphase spreads (*n* = 3) were analyzed and quantified. Statistical significance was calculated with unpaired *t*-test. * *p* value ≤ 0.05, ** *p* value ≤ 0.01, **** *p* value ≤ 0.0001. Whole-cell lysates of the same cells were subjected to immunoblotting.

Thus far, our data suggested that elevated replication stress is the major source of DNA damage arising in CtIP/BARD1 double-deficient cells. To further define the consequences of CtIP and BARD1 depletion on replication dynamics, we conducted a dual-labeling DNA fiber assay, comparing the tract lengths of sister forks when DNA synthesis is challenged with hydroxyurea (HU), a potent inhibitor of the enzyme ribonucleotide reductase. Intriguingly, CtIP or BARD1 single knockdown led to sister fork asymmetry that was further increased upon co-depletion of both factors (Figure 5A), indicating that individual replicons are more susceptible to persistent stalling under these conditions.

Earlier we observed progressive ATM signaling upon combined disruption of CtIP and BARD1 even in otherwise undamaged cells (Figure S2C), indicative of DSB formation. To directly assess whether DSBs might be a consequence of unrepaired lesions accumulating in response to replication stress, we performed pulsed field gel electrophoresis (PFGE) analysis on genomic DNA extracted from HU-treated cells. Indeed, simultaneous disruption of both proteins resulted in elevated DSB formation, whereas single knockdown of CtIP or BARD1 did not cause any significant increase in DNA breaks formation compared to control cells (Figure 5B). Persisting DSBs arising from replication stress have the potential to cause chromosomal instability, particularly in the context of HR deficiency [52]. To this end, we analyzed metaphase spreads prepared from HU-treated cells. Consistent with our previous findings, co-depletion of CtIP and BARD1 further elevated the frequency of chromosomal aberrations per metaphase compared to single knockdowns (Figure 5C). Of note, we predominantly observed chromosomal fragments and deletions that can be the result of chromosome or chromatid breakage (Figure 5C). Taken together, our data indicated the non-overlapping roles of CtIP and BARD1 in facilitating the progression of challenged replication forks and counteracting replication stress-induced DSBs. As they cannot be fixed by HR, chromosome fragments and fusions accumulate over time, likely accounting for the synthetic sick phenotype observed in CtIP/BARD1-deficient cells.

## 4. Discussion

CtIP plays an ambiguous role in tumorigenesis that can be intricately linked with tumor type. On one side it has been postulated that CtIP can function as a tumor suppressor. Along this line, CtIP germline variants have been recently identified in Danish cohorts of breast cancer patients negative for pathogenic mutations of BRCA1 and BRCA2 [53]. In addition, CtIP+/**−** heterozygous mice develop multiple tumors, mainly large B-cell lymphomas [54]. However, this finding has been challenged by a more recent study showing how a single null allele of CtIP does not impact tumor-free survival in mice [15]. Moreover, in the same study, it has been observed that mammary-specific inactivation of CtIP rather decreases tumorigenesis in p53 null mice. The latter suggests an oncogenic activity of CtIP, which can be related to its role in alternative-end joining (a-EJ), a repair pathway that leads to chromosomal translocations and genome instability [55]. Interestingly, *CtIP* is amplified in pancreatic cancers and high expression levels are associated with poor prognosis. Despite its controversial role in tumorigenesis, CtIP has a prognostic value in different tumors and impaired CtIP activity can be associated with a better response to chemotherapy [56]. 

Here, through an image-based high-content RNA interference (RNAi) screen, we identified an SSL interaction between *CtIP* and *BARD1*, a low-to-moderate breast cancer risk gene, whose mutations have been associated with the development of many types of tumors [57]. 

CtIP is a phosphorylation-dependent binding partner of the BRCA1 BRCT domains [10]. CtIP transiently interacts with the BRCA1-BARD1 complex in the G2 phase and participates in the activation of the G2/M transition checkpoint [10]. CtIP binding to BRCA1 has also been implicated in transcriptional regulation and it suppresses p21 transactivation [58]. Moreover, the CtIP-BRCA1 interaction was found to be critical for the initiation of transcription-associated HR repair of DSBs [59] and to accelerate DNA-end resection [60]. On the other hand, the BRCA1-BARD1 complex can indirectly promote CtIP-mediated resection and HR by counteracting 53BP1 chromatin accumulation [51]. Finally, although the CtIP-BRCA1 interaction is dispensable for fork protection, we have recently revealed that the two proteins act in a synergistic manner to protect nascent DNA strands from nucleolytic degradation [9]. Here, we corroborate and extend this finding by showing that CtIP establishes a synthetic lethal interaction with BARD1, the obligate partner of BRCA1, due to their role in preserving replication fork integrity. Our data support a model in which CtIP and BARD1 are interconnected through a synthetic sick genetic interaction that can be confined to the S-phase and does not occur in G2, when the CDK1-dependent phosphorylation of CtIP on S327 promotes its association with BRCA1. 

In the combined absence of BARD1 and CtIP, disproportionate levels of endogenous DNA lesions arise in the context of replication and cannot be appropriately addressed due to simultaneous dysfunction of the HR pathway. Therefore, failure to resolve replication intermediates results in persisting DNA lesions that may cause chromosomal aberrations, a major source of genome instability.

Our data are consistent with the hypothesis that endogenous DNA damage can conceivably arise as a result of enhanced replication stress increasing the level of DNA breakage and chromosomal aberrations [52]. Importantly, if DNA damage exceeds the repair capacity, programmed cell death will be initiated [61]. In rare cases however, such aberrations may escape from cell surveillance pathways and contribute to mutation and malignant transformation. Importantly, it has been proposed that dosage insufficiencies of DNA repair genes might be initially tolerated by a cell but become unmasked when DNA perturbations accumulate to reach a certain threshold [62]. Further support for this concept came from findings that innate haploinsufficiency for BRCA1 leads to cell-type-specific genomic instability as well as impaired proficiency of primary cells to respond to replication stress; meanwhile, DSB repair was hardly compromised [63,64]. This illustrates how intrinsically reduced levels of key DDR proteins can contribute to a phenotype of mild but persistent genotoxic stress that may initially be tolerated by cell surveillance mechanisms but that may ultimately cause chromosomal instability and tumorigenesis. 

Interestingly, the genetic interaction between CtIP and BARD1 occurs independently of the formation of the BRCA1-BARD1 complex. BARD1 can establish BRCA1-independent interactions and be involved in different pathways of oncogenesis. Moreover, BRCA1 RING domain mutations defective of the BARD1 heterodimerization display PARP1 inhibitors and platinum resistance [65,66]. Therefore, we envision a scenario where the CtIP-BARD1 genetic interaction could potentially be exploited therapeutically for cancer treatment especially in the context of BRCA1-deficient cancers that develop therapy resistance.

## 5. Conclusions

Our study reveals that CtIP genetically interacts with BARD1 to promote faithful DNA replication, underscoring a previously underestimated role of the BRCA1-C complex in combating replication stress-induced genome instability. In addition, we decipher further details of CtIP action in constraining endogenous DNA replication stress, highlighting its significance for averting tumorigenesis. Overall, our study corroborates the idea of CtIP as a novel therapeutic target for the treatment of the BARD1/BRCA1-deficient tumors.

## Data Availability

Data presented in this study are available on request from the corresponding authors.

## Supplementary Materials

The following are available online at https://www.mdpi.com/article/10.3390/cells11040643/s1, Figure S1. Identification of a synthetic sick genetic interaction between CtIP and BARD1 through RNAi image-based screening. Figure S2. Concomitant loss of CtIP and BARD1 triggers apoptosis by DNA damage. Figure S3. CtIP and BARD1 deficiency induces 53BP1 foci formation indicative of HR deficiency. Table S1. List of primary antibodies. Table S2. List of siRNA oligos. Table S3. List of DNA primers. Table S4. Analysis of siRNA screen results.
