# Supplementary material for: RNAi Screening Uncovers a Synthetic Sick Interaction between CtIP and the BARD1 Tumor Suppressor
Source: Cells. 2022 Feb 12;11(4):643. doi: 10.3390/cells11040643 (PMC8870135; doi:10.3390/cells11040643)
Supplement: Supplementary file 1 [file cells-11-00643-s001.zip › supplementary Materials.pdf]

Supplementary Material

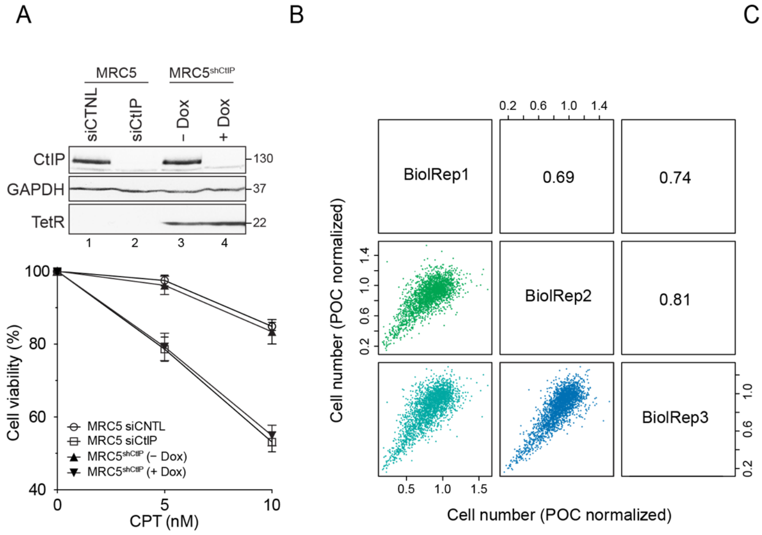

C

Gene	Gene ID	Rank	RSA p-value
LMO4	8543	1	0.0019
MRE11A	4361	2	0.0055
ESCO1	114799	3	0.0078
CDC25A	993	4	0.0116
DDB2	1643	5	0.0126
E2F6	1876	6	0.0149
TP53	7157	7	0.0152
BARD1	580	8	0.0175
E2F3	1871	9	0.0193
SLX4	84464	10	0.0245
CDC6	990	11	0.0290
KAT5	10524	12	0.0374
CHFR	5573	13	0.0386
EXO1	9156	14	0.0392
RAD54L	8438	15	0.0440
LIG1	3978	16	0.0451
RAD18	56852	17	0.0460
POLQ	10721	18	0.0486
CTBP2	1488	19	0.0509
LIG3	3980	20	0.0521
BRCA1	672	21	0.0550

Supplementary Figure S1. Identification of a synthetic sick genetic interaction between CtIP and BARD1 through RNAi image-based screening. **(A)** MRC5 cells were transfected with the indicated siRNAs for 48 h. MRC5^{shCtIP} or MRC5^{shLacZ} cells were cultivated in the absence or presence of Dox (1 μg/mL) for 48 h. Subsequently, cells were treated with the indicated doses of CPT. Survival was determined after 4 days using the CellTiter-Blue® cell viability assay. Data are presented as mean ± s.e.m. (n = 5). Whole cell lysates were prepared and subjected to immunoblotting. **(B)** Replicate correlation plots to visualize overall reproducibility of the RNAi screens. Each data point represents a cell number obtained from one well. Pearson correlation coefficients between three biological replicates are displayed. **(C)** Candidate genes for SSL interactions with CtIP were ranked by redundant siRNA activity (RSA) analysis. A RSA p-value cut-off of p<0.05 was defined to determine a hit list.

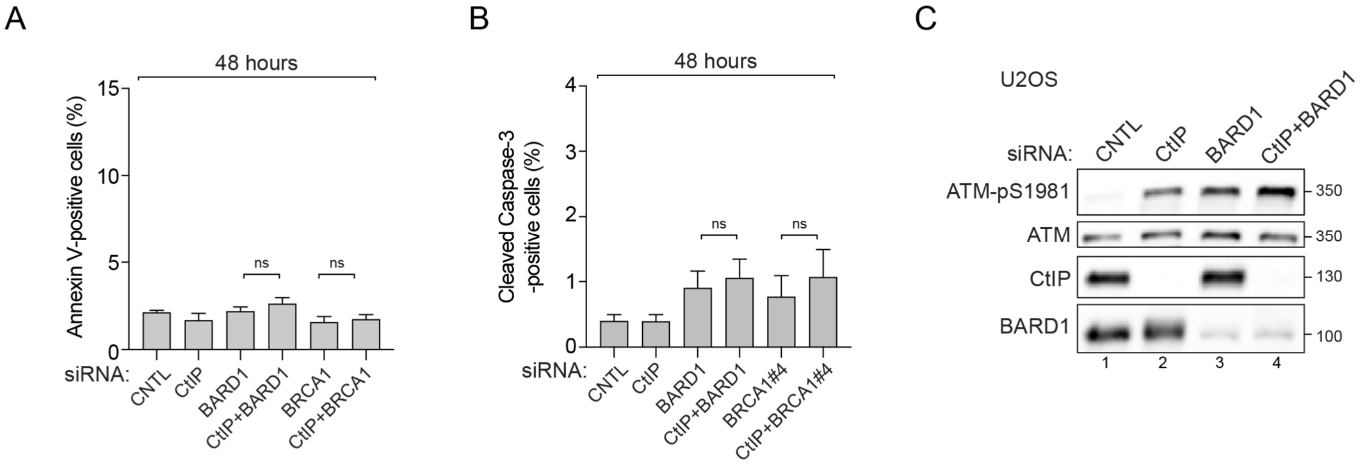

Supplementary Figure S2. Concomitant loss of CtIP and BARD1 triggers apoptosis by DNA damage. (**A,B**) U2OS cells transfected with the indicated siRNA for 48 h were harvested and induction of apoptosis was determined by annexin V (A) or cleaved caspase-3 (B) after 48 h. Statistical significance was calculated with unpaired *t*-test. ^{ns} P value > 0.05. Representative images are shown. Whole-cell extracts of corresponding samples were analyzed by western blotting. (**C**) U2OS cells transfected with the indicated siRNA for 48 h were harvested for western blot analysis with the indicated antibodies.

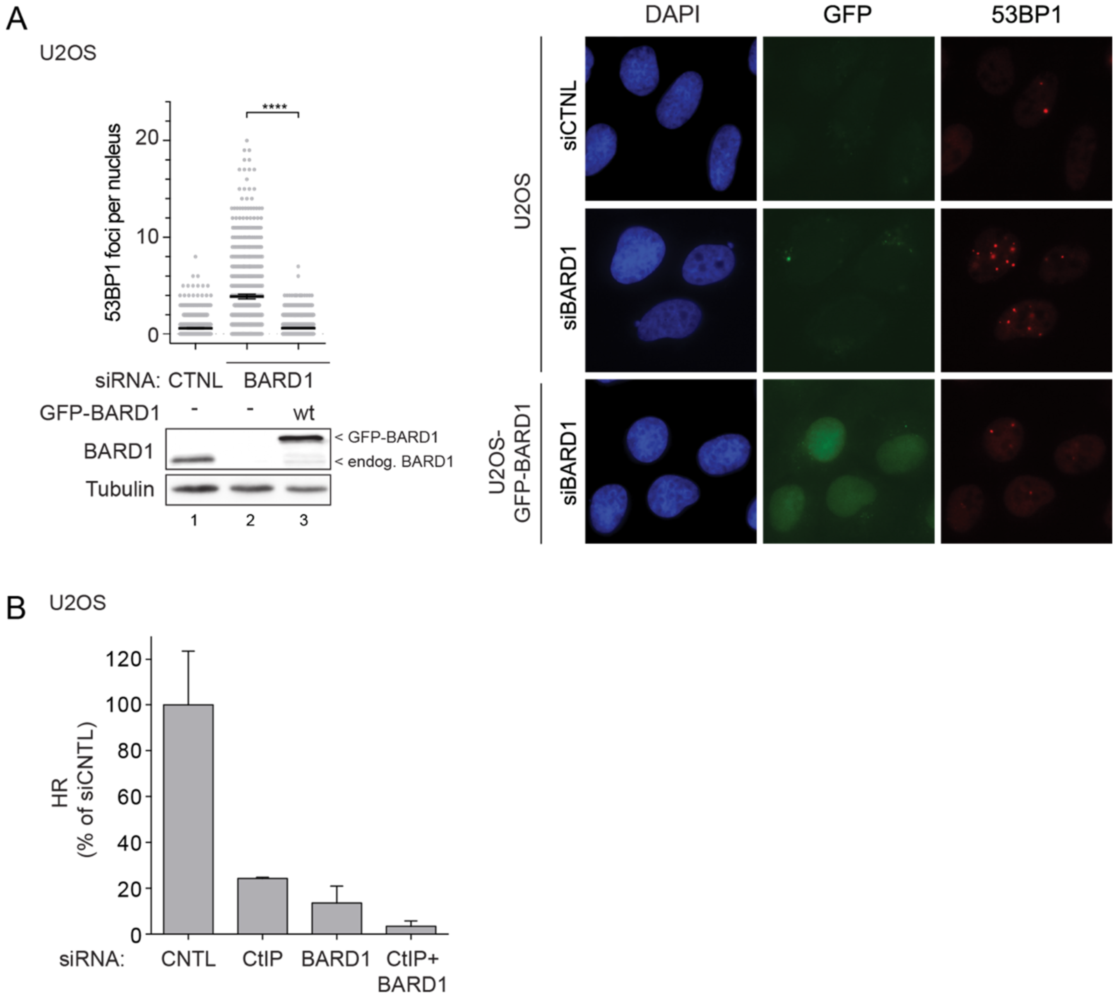

Supplementary Figure S3. CtIP and BARD1 deficiency induces 53BP1 foci formation indicative of HR deficiency (**A**) Parental U2OS cells and U2OS cells stably expressing siRNA-resistant GFP-tagged human BARD1-wt were transfected with siBARD1 for 48 h, and immuno-fluorescence was performed using anti-53BP1 antibodies. Scatter plots represent quantification of 53BP1 nuclear bodies per nucleus. Black bars denote mean ± 95 % confidence interval assessed in at least 600 nuclei per knockdown condition (n = 3). Statistical significance was calculated with Kolmogorov-Smirnov test. **** P value ≤ 0.0001. Representative images are shown. Whole-cell

extracts of corresponding samples were analyzed by western blotting. (**B**) U2OS cells containing a classical GFP-based DNA repair reporter for HR were transfected with the indicated siRNAs and six hours later transfected with the *I-SceI* expression plasmid and the exogenous *GFP* donor template. Cells were harvested 72 h after siRNA transfection and GFP-positive cells indicating HR repair activity were assessed by flow cytometry. Data are presented as mean ± s.e.m. (n = 2).

Table S1: Primary antibodies

Antibody target	Species	Supplier/Reference	Applications*
53BP1	mouse	Millipore (MAB3802)	QIBC
53BP1	rabbit	Abcam (ab21083)	IF
ATM-pS1981	rabbit	Abcam (ab81292)	IB
ATM (2C1)	mouse	GeneTex (GTX70103)	IB
BARD1	rabbit	[1]	IB and IF
BRCA1 (D9)	mouse	Santa Cruz	IB
BRCA1 (Ab-1)	mouse	Calbiochem/Merck	IB
BrdU/IdU (B44)	mouse	BD Biosciences (347580)	DNA Fibers
BrdU/CldU	rat	Abcam (ab6326)	DNA Fibers
Cleaved Caspase-	rabbit	Cell Signaling (9661)	FACS
CtIP (D4)	mouse	Santa Cruz	IB
CtIP (14-1)	mouse	Active Motif (61141)	IB
CtIP-pS327	rabbit	[2]	IB
Cyclin A (6E6)	mouse	Abcam (ab16726)	IF
FLAG (M2)	mouse	Sigma-Aldrich (F3165)	IB
GAPDH (6C5)	mouse	Millipore/Merck (MAB374)	IB
GFP	rabbit	Abcam (ab290)	IB
HA	rabbit	Abcam (ab9110)	IB
γH2AX (20E3)	rabbit	Cell Signaling (9718)	IF and FACS
TetR (9G9)	mouse	Clontech/Takara (631131)	IB
Tubulin (DM1A)	mouse	Sigma Aldrich (T9026)	IB

*IB: Immunoblot, IF: Immunofluorescence

Table S2: siRNA oligos

Name	Sense sequence (5'-3')	Reference/Cat. No.	Supplier
CNTL	Negative Control No. 2 siRNA	4390846	Ambion
CtIP	GCUAAAACAGGAACGAAUC	[3]	Ambion

BARD1#1 (=BARD1)	GCCUGUCGAUUAUACAGAU	s1885	Ambion
BARD1#2	GCAGUAAUUCUUAAGGCUA	s1886	Ambion
BARD1#3	CGCUAUUGCUGCUACCAGA	s1887	Ambion
BARD1#4	CUGAAUAUUAUACCAGAUGAA	this work	Ambion
BARD1#JM	UGGUUUAGCCCUCGAAGUAAG	[4]	Sigma
BRCA1#2	CAUGCAACAUAACCUGAUA	s459	Ambion
BRCA1#3 (=BRCA1)	CAGCUACCCUUCCAUCAUA	s458	Ambion
BRCA1#4	GGAACCUGUCUCCACAAAG	[5]	Ambion

Table S3: DNA primers

**Name**	**Sense sequence (5′-3′)**
BARD1_siRes_for	CTGCGGCCTGTCGACTACACGGATGATGAAAGTATGAAATCGCTATTGC
BARD1_siRes_rev	ACTTTCATCATCCGTGTAGTCGACAGGCCGCAGACCAAATATATTAAC
BARD1_clon_for	CGATCGATATCCCGGATAATCGGCAGCCGAGG
BARD1_clon_rev	ATAGATAGCGGCCGCTCAGCTGTCAAGAGGAAGCAA
BARD1_seq1_for	ATGCCGGATAATCGGCAGCC
BARD1_seq2_for	CATCCTCAGCTAGCCACTGC

References:

1. Wu LC, Wang ZW, Tsan JT, Spillman MA, Phung A, Xu XL, Yang MC, Hwang LY, Bowcock AM, Baer R (1996) Identification of a RING protein that can interact in vivo with the BRCA1 gene product. *Nat Genet* **14**: 430–440.
2. Barton O, Naumann SC, Diemer-Biehs R, Künzel J, Steinlage M, Conrad S, Makharashvili N, Wang J, Feng L, Lopez BS, et al. (2014) Polo-like kinase 3 regulates CtIP during DNA double-strand break repair in G1. *The Journal of Cell Biology* **206**: 877–894.
3. Sartori AA, Lukas C, Coates J, Mistrik M, Fu S, Bartek J, Baer R, Lukas J, Jackson SP (2007) Human CtIP promotes DNA end resection. *Nature* **450**: 509–514.

4. Daza-Martin M, Starowicz K, Jamshad M, Tye S, Ronson GE, MacKay HL, Chauhan AS, Walker AK, Stone HR, Beesley JFJ, et al. (2019) Isomerization of BRCA1-BARD1 promotes replication fork protection. *Nature* **571**: 521–527.
5. Bruun D, Folias A, Akkari Y, Cox Y, Olson S, Moses R (2003) siRNA depletion of BRCA1, but not BRCA2, causes increased genome instability in Fanconi anemia cells. *DNA Repair (Amst)* **2**: 1007–1013.
